# The Rhythm of Risk: Sexual Behaviour, PrEP Use and HIV Risk Perception Between 1999 and 2018 Among Men Who Have Sex with Men in Amsterdam, The Netherlands

**DOI:** 10.1007/s10461-020-03109-4

**Published:** 2020-12-02

**Authors:** Maartje Basten, Chantal den Daas, Janneke C. M. Heijne, Anders Boyd, Udi Davidovich, Ganna Rozhnova, Mirjam Kretzschmar, Amy Matser

**Affiliations:** 1 Julius Center for Health Sciences and Primary Care, University Medical Center Utrecht and Utrecht University, Internal Mail No Str 6.131, P.O. Box 85500, 3508 GA Utrecht, The Netherlands; 2 Department of Infectious Diseases, Research and Prevention, Public Health Service of Amsterdam, Amsterdam, The Netherlands; 3 Center for Infectious Diseases Control, National Institute for Public Health and the Environment, Bilthoven, The Netherlands; 4 Aberdeen Health Psychology Group, Institute of Applied Health Sciences, Aberdeen, Scotland; 5 Stichting HIV Monitoring, Amsterdam, The Netherlands; 6 BioISI—Biosystems & Integrative Sciences Institute, Faculdade de Ciências, Universidade de Lisboa, Lisbon, Portugal

**Keywords:** HIV risk perception, Sexual behaviour, Pre-exposure prophylaxis, Sexually transmitted diseases, Men who have sex with men, Cohort studies

## Abstract

HIV risk perception plays a crucial role in the uptake of preventive strategies. We investigated how risk perception and its determinants changed between 1999 and 2018 in an open, prospective cohort of 1323 HIV-negative men who have sex with men (MSM). Risk perception, defined as the perceived likelihood of acquiring HIV in the past 6 months, changed over time: being relatively lower in 2008–2011, higher in 2012–2016, and again lower in 2017–2018. Irrespective of calendar year, condomless anal intercourse (AI) with casual partners and high numbers of partners were associated with higher risk perception. In 2017–2018, condomless receptive AI with a partner living with HIV was no longer associated with risk perception, while PrEP use and condomless AI with a steady partner were associated with lower risk perception. We showed that risk perception has fluctuated among MSM in the past 20 years. The Undetectable equals Untransmittable statement and PrEP coincided with lower perceived risk.

**Electronic supplementary material:**

The online version of this article (10.1007/s10461-020-03109-4) contains supplementary material, which is available to authorized users.

## Introduction

Current HIV risk reduction strategies include condom use, maintaining undetectable viral load (for individuals living with HIV), regular HIV testing, and use of pre-exposure prophylaxis (PrEP). These strategies are effective means of reaching the goal of zero HIV infections among men who have sex with men (MSM). Recent research showed that MSM who had a higher perceived risk of acquiring HIV were more interested in taking PrEP [1–3], showed higher PrEP adherence [4], and were more likely to get tested for HIV [5, 6]. Conversely, unrealistically low risk perception was found to be an important barrier for successful PrEP implementation [7] and HIV testing [8, 9], and was one of the main reasons why patients with late HIV diagnosis did not get tested earlier [10]. These studies show that in order for MSM to take preventive measures, a realistic perception of HIV risk is crucial. To improve uptake of preventive strategies, further understanding is needed as to what factors influence risk perception.

Sexual behaviour and other risk factors for acquiring HIV, including drug use and history of sexually transmitted infections (STI), have been found to be associated with higher risk perception in MSM, although specific findings have been mixed [1, 7, 11, 12]. For example, Wilton et al. found that high risk perception was associated with past history of bacterial STI and a high score on the HIRI-MSM, an HIV risk screening tool based on number of partners, frequency of condomless anal intercourse (AI) and use of drugs [7]. Biello et al. found that high risk perception was associated with a high number of receptive AI partners, but not with an STI diagnosis in the past year [1]. Kesler et al. found that high risk perception was associated with low condom use with a regular partner living with HIV and with use of poppers, but, in contrast to Wilton et al. [7], was not associated with high HIV risk (i.e. high score on the HIRI-MSM) [11]. These inconsistent findings may be explained by different definitions of risk perception, i.e. perceived risk of having acquired HIV [12], current perceived risk [1, 7] or perceived risk to acquire HIV in the future [11]. Also, differences in sexual behaviour measures and study population characteristics may have played a role. Importantly, these studies were cross-sectional and did not take into account possible longitudinal changes in risk perception and the mutual relationship with sexual behaviour. Little is known about how risk perception among MSM has evolved in the past decades, especially in light of the developments in treatment and prevention, fluctuations in HIV incidence, and changes in sexual behaviour within the population.

Since the introduction of ART in 1996, MSM in Western countries have been more likely to have condomless AI [13, 14] and to have more casual sex partners [15, 16]. Risk behaviours have continued to increase [17, 18] and have been accompanied by increasing STI incidence [15, 18, 19]. From 2000 onwards, the number of new HIV diagnoses increased among MSM in Western countries [20, 21], yet, in the past decade, numbers of diagnosed HIV infections have been decreasing in the Netherlands [18, 22], most likely due to increased testing and early treatment. The evolution of sexual behaviour, STI and HIV incidence, and HIV treatment and prevention may have affected perceived risk to acquire HIV among MSM and in turn, could influence the willingness of MSM to partake in preventive measures.

The aims of the current study were to examine (1) the course of HIV risk perception among HIV-negative MSM over the past 20 years, and (2) how sexual behaviours, recent history of STI, and PrEP use are related to risk perception and whether these associations changed over time. Risk perception was defined as the perceived likelihood of acquiring HIV in the past 6 months. We aimed to study several behavioural characteristics, including partner type, numbers of partners, condom use, chemsex, and AI during group sex. This study was performed using longitudinal data from the Amsterdam Cohort Studies (ACS) among MSM collected between 1999 and 2018.

## Methods

### Study Population and Procedure

The ACS among MSM started in 1984 in Amsterdam, the Netherlands, and is an open, ongoing prospective cohort study to investigate the epidemiology, psychosocial determinants, course of infection, and pathogenesis of HIV [23]. The ACS was approved by the Medical Ethics Committee of the Academic Medical Center, University of Amsterdam, Amsterdam, the Netherlands (MEC 07/182). Participation was voluntary and written informed consent was obtained from every participant at intake. Men were eligible for participation if they lived in or around Amsterdam and had sex with other men in the 6 months prior to recruitment (see also [13, 18]). Recruitment was done by “convenience sampling” (e.g. brochures at the STI clinic, advertisements in the gay scene) and “chain referral sampling” (participants recruited by other participants). Recruitment was limited to young MSM under 30 years of age during several time periods, to prevent aging of the cohort. All participants visited the Public Health Service of Amsterdam every 6 months to complete a self-administered questionnaire about their sexual behaviour and related psychosocial determinants and to get tested for HIV and, since October 2008, for other STI (see also [13, 18]). Participants were informed about positive test results after their visit.

The present study comprised MSM who visited the ACS at least once between 1 January 1999 and 31 December 2018, including 40 study waves. MSM were included in the present study if they were HIV negative at the start of the study period and had at least one measure of risk perception. Follow-up continued until HIV-positive diagnosis, last ACS visit, death, or 31 December 2018.

### Risk Perception

At the beginning of each questionnaire, MSM were asked to rate the likelihood that they acquired HIV in the preceding 6 months, defined herein as their “perceived HIV risk”. This question was answered on a 7-point Likert scale from 1 being ‘impossible’ to 7 being ‘very likely’. Because few MSM responded with high perceived risk, we combined answer categories 5 through 7 into one category, resulting in 5 levels of risk perception.

### Sexual Behaviour, Recent STI and PrEP Use

At every visit, questions were asked about sexual behaviour with steady and casual partners in the preceding 6 months. The following indicators of sexual behaviour were available from the second half of 1999 to 2018: numbers of casual partners with whom participants had insertive AI and receptive AI, condomless AI with a casual partner (yes/no), and condomless AI with a steady partner, which was categorized into (1) no steady partner, (2) no condomless AI with steady partner, and (3) condomless AI with a steady partner. From 2002 onwards, condomless receptive AI with a steady or casual partner living with HIV (yes/no) was assessed. From 2008 onwards information was available on AI during group sex (yes/no) and chemsex (yes/no). Chemsex was defined as using GBL, GHB, mephedrone, methamphetamine, ketamine, amphetamine, cocaine, or XTC during sex.

From 2008 onwards, recent STI was defined as a bacterial STI reported or tested at the previous ACS visit maximum 1 year ago (yes/no). This included self-reported gonorrhoea, chlamydia, or syphilis diagnosis in the past 6 months or a positive test result for oral, anal, or urethral gonorrhoea and chlamydia or syphilis (tests were performed routinely from October 2008 onwards).

PrEP use in the past 6 months (yes/no) was based on self-report and was available from the second half of 2015 onwards [24].

Over time, some modifications were made in behaviour questionnaires, but variables under study were assessed consistently over time (see Supplementary Table S1 for a description).

### Covariates

Age at visit, calendar year of first visit between 1999 and 2018, and educational level were taken into account to study change in risk perception. Educational level was assessed at intake and was considered ‘high’ with completion of higher vocational training or university, and ‘middle and low’ with completion of secondary vocational training, high school, basic vocational education, or primary school.

### Statistical Analyses

Longitudinal change in risk perception between 1999 and 2018 was studied using multilevel ordinal logistic regression analysis. Models included a random intercept to account for multiple observations nested within each participant. In the model, risk perception was treated as an ordinal outcome variable. Calendar time in half-years was treated as a predicting variable. We examined the relative differences in risk perception score between each half-year increment versus the grand mean, that is the distribution of the mean prevalence of each risk perception score across all time points. This model calculates an odds ratio that can be interpreted as the average odds of scoring one-point higher on the risk perception scale (the average of scoring 1 versus 2–5; 1–2 versus 3–5; 1–3 versus 4–5; 1–4 versus 5). Using the grand mean as a reference group allows us to demarcate periods of increased and decreased risk perception compared to what would be expected over the entire calendar period. The analysis was adjusted for age, year of study entry, and educational level. Based on these half-year differences in risk perception compared to the grand mean, we defined periods of relatively low and relatively high risk perception. To examine whether changes in risk perception were not due to MSM entering or leaving the cohort, sensitivity analyses were performed among participants who were in the cohort for a longer time period (> 6 years). We also repeated our analysis among participants who did not use PrEP to understand its contribution to change in risk perception.

To examine how sexual behaviours in the past 6 months, recent STI, and PrEP use in the past 6 months were related to risk perception and whether these relations changed over time, we again used multilevel ordinal logistic regression analysis with risk perception as an ordinal outcome. Associations with behaviours, recent STI and PrEP use were examined within periods of relatively low and high risk perception, which we based on the analyses described above. Univariable and multivariable analyses were performed in each period to examine the individual contribution of each predictor to risk perception. Multivariable analyses were adjusted for age and educational level. The first model included behaviours which were assessed between second half of 1999 and 2018: numbers of receptive and insertive AI casual partners, having condomless AI with a casual partner, having condomless AI with a steady partner, and having condomless receptive AI with a partner living with HIV. The second model was limited to data from 2008 onwards and additionally included the variables recent STI, chemsex, and AI during group sex. The third model additionally included PrEP use, which was available from second half of 2015 onwards. For all models, we tested the proportional odds assumption using a likelihood ratio test and the Brant test. Analyses were performed using STATA Intercooled 15.1 (STATA Corporation, College Station, Texas, USA).

## Results

### Longitudinal Course of Risk Perception

**Fig. 1:**
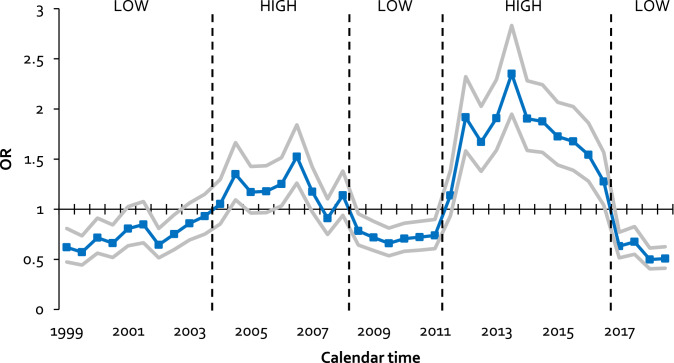
Relative differences in risk perception score across time among 1309 MSM between 1999 and 2018 (17,811 visits). Relative differences are presented as OR’s for each wave compared to the grand mean (i.e. the distribution of the risk perception score across all visits). OR’s below one indicate lower odds for a one-point higher score on the risk perception scale, while OR’s above one indicate higher odds for a one-point higher score on the risk perception scale. OR’s are adjusted for age, year of study entry and education level. Lines in grey define 95% confidence intervals. Dashed vertical lines distinguish periods of relatively lower and higher risk perception scores for the study population

The total sample consisted of 1323 MSM with 17,870 visits (A flowchart of the study sample is provided in Supplementary Fig. S1). Sample characteristics at inclusion are described in Table 1. Across waves, the percentage of MSM who considered their likelihood of acquiring HIV as ‘impossible’ ranged between 21 and 40% and as ‘substantial’ to ‘very likely’ (score 4 or 5–7) between 3 and 12% (Supplementary Fig. S2). Figure 1 shows that risk perception followed a sinusoidal trend and was significantly higher and lower than the grand mean at several consecutive time points. From 1999 to 2000 and in 2002 risk perception was significantly lower than the grand mean. In the second half of 2004 and second half of 2006 it was significantly higher, while in the second half of 2008 until the first half of 2011 risk perception was again lower. In 2012 until the first half of 2016 risk perception was significantly higher and in 2017 and 2018 risk perception was again lower than the grand mean. Based on these differences, we were able to distinguish five periods of relatively low or high risk perception (Fig. 1): (1) 1999–2003: low risk perception; (2) 2004–first half of 2008: high risk perception; (3) second half of 2008–first half of 2011: low risk perception; (4) second half of 2011–2016: high risk perception; and (5) 2017–2018: low risk perception. Sensitivity analyses showed the same longitudinal patterns among participants with more than 6 years in the cohort, covering at least 2 of these risk perception periods (n = 570 MSM; 14,083 visits), and among non-PrEP users in 2017–2018 (n = 677 MSM; 1971 visits; Supplementary Fig. S3).

**Table 1:** Sample characteristics

	N = 1323
Number of visits, *Mdn* (IQR)	9 (3–22)
Migration background, n (%)	
Dutch	839 (80.5%)
First generation migrant	166 (15.9%)
Second generation migrant	37 (3.6%)
Education, n (%) high	950 (72.6%)
Age^a^, *M* (SD)	31.3 (9.9)
Age sexual debut, *M* (SD)	18.1 (4.2)
HIV seroconversions during follow-up, n (%)	117 (8.8%)
Sexual behaviour and partnership in past 6 months^a^	
Steady partnership, n (%)	832 (63.1%)
Condomless AI with steady partner, n (%)	487 (37.8%)
Number of casual insertive AI partners, *M* (SD)/*Mdn* (IQR)	3.1 (8.1)/1 (0–3)
Number of casual receptive AI partners, *M* (SD)/*Mdn* (IQR)	3.1 (8.5)/0 (0–2)
Condomless AI with casual partner, n (%)	340 (26.0%)

Percentages of missing data were: Migration background 21.2%; education 1.1%; age sexual debut 2.9%; sexual behaviour and partnership variables: 0.4–2.6%

*AI* anal intercourse

^a^Reported on participant’s first visit between 1999 and 2018

### Sexual Behaviours and Risk Perception

Table 2 shows univariable associations between sexual behaviours, recent STI, and PrEP use and risk perception within these five periods of relatively low and high perceived risk. All factors were associated with perceived risk in one or more time periods.

**Table 2:** Univariable associations between sexual behaviours, STI and PrEP use and risk perception within periods of relatively low and high risk perception

	1999(2)–2003^a^ Low risk perception n visits = 3357		2004–2008(1)^a^ High risk perception n visits = 3912		2008(2)–2011(1)^a^ Low risk perception n visits = 2474		2011(2)–2016^a^ High risk perception n visits = 5549		2017–2018 Low risk perception n visits = 2243	
	*Mdn* (IQR) /%	OR (95% CI)	*Mdn* (IQR) /%	OR (95% CI)	*Mdn* (IQR) /%	OR (95% CI)	*Mdn* (IQR) /%	OR (95% CI)	Mdn (IQR) /%	OR (95% CI)
Number of casual partners insertive AI^b^	0 (0–2)	2.44 (2.11; 2.82)	0 (0–2)	2.42 (2.14; 2.73)	0 (0–3)	1.97 (1.68; 2.32)	1 (0–4)	1.90 (1.75; 2.07)	1 (0–5)	1.77 (1.54; 2.03)
Number of casual partners receptive AI^b^	0 (0–1)	2.56 (2.21; 2.97)	0 (0–2)	2.42 (2.13; 2.74)	0 (0–2)	2.20 (1.86; 2.61)	0 (0–2)	1.92 (1.75; 2.10)	0 (0–3)	1.69 (1.46; 1.94)
Condomless AI with casual partner	17.4%	6.94 (5.43; 8.88)	18.5%	6.25 (5.02; 7.77)	20.5%	4.12 (3.07; 5.52)	27.2%	4.59 (3.91; 5.39)	34.5%	4.08 (3.08; 5.40)
No condomless AI with steady partner^c^	25.3%	0.82 (0.64; 1.05)	23.3%	0.67 (0.53; 0.85)	25.3%	0.52 (0.36; 0.73)	25.2%	0.46 (0.37; 0.57)	23.8%	0.50 (0.34; 0.73)
Condomless AI with steady partner^c^	40.5%	0.75 (0.59; 0.95)	40.7%	0.65 (0.52; 0.80)	38.8%	0.64 (0.46; 0.89)	38.7%	0.43 (0.36; 0.52)	38.8%	0.41 (0.30; 0.58)
Condomless receptive AI with partner living with HIV	–	–	1.5%	8.83 (4.23; 18.46)	2.0%	6.57 (2.87; 15.04)	3.4%	2.66 (1.79; 3.95)	8.6%	1.16 (0.72; 1.89)
AI during group sex					21.6%	2.26 (1.67; 3.08)	24.4%	1.70 (1.43; 2.01)	23.3%	1.86 (1.37; 2.51)
Chemsex					20.1%	2.30 (1.63; 3.25)	25.4%	1.85 (1.53; 2.23)	33.8%	1.49 (1.09; 2.04)
Recent STI					9.2%	1.03 (0.72; 1.50)	12.2%	1.57 (1.29; 1.91)	14.8%	1.05 (0.74; 1.49)
PrEP use									12.0%	0.38 (0.24; 0.58)

*AI* anal intercourse, *STI* sexually transmitted infection, *PrEP* pre-exposure prophylaxis

^a^(1) and (2) indicate first and second wave within the year respectively

^b^Numbers of casual partners are log transformed in the model

^c^Reference group is ‘no steady partner’

The first multivariable model, only including all behaviours assessed during the whole study period (Table 3), showed that higher number of casual partners with whom participants had insertive and receptive AI and having condomless AI with a casual partner were associated with higher perceived risk across all time periods. Having a steady partner and having condomless AI with a steady partner were also significantly associated with higher risk perception between 1999 and 2003 (aOR 1.35 [95% CI 1.04–1.76] and 1.54 [95% CI 1.18–2.00]). In contrast, these factors were associated with lower risk perception from 2011 onwards (range aOR 0.63–0.80). Having condomless AI with a partner living with HIV was significantly associated with higher risk perception between 2004 and 2016 (range aOR 1.89–7.17), but this association was no longer found in 2017–2018.

**Table 3:** Multivariable associations between sexual behaviours and risk perception within periods of relatively low and high risk perception between 1999 and 2018

	1999(2)–2003^a^ Low risk perception	2004–2008(1)^a^ High risk perception	2008(2)–2011(1)^a^ Low risk perception	2011(2)–2016^a^ High risk perception	2017–2018 Low risk perception
	n visits = 3137	n visits = 3614	n visits = 2269	n visits = 5373	n visits = 2152
	OR (95% CI)	OR (95% CI)	OR (95% CI)	OR (95% CI)	OR (95% CI)
Number of casual partners insertive AI^b^	1.48 (1.24; 1.76)	1.60 (1.39; 1.84)	1.36 (1.14; 1.64)	1.31 (1.19; 1.45)	1.31 (1.11; 1.54)
Number of casual partners receptive AI^b^	1.78 (1.49; 2.12)	1.60 (1.38; 1.85)	1.61 (1.33; 1.94)	1.33 (1.20; 1.47)	1.24 (1.05; 1.47)
Condomless AI with casual partner	4.66 (3.54; 6.14)	3.96 (3.11; 5.03)	2.75 (1.99; 3.81)	2.98 (2.50; 3.56)	2.79 (2.01; 3.88)
No condomless AI with steady partner^c^	1.35 (1.04; 1.76)	1.10 (0.86; 1.41)	0.83 (0.58; 1.19)	0.69 (0.56; 0.86)	0.80 (0.54; 1.19)
Condomless AI with steady partner^c^	1.54 (1.18; 2.00)	1.17 (0.92 1.48)	1.08 (0.76; 1.54)	0.72 (0.59; 0.87)	0.63 (0.45; 0.88)
Condomless receptive AI with partner living with HIV	–	7.17 (3.26; 15.76)	3.81 (1.58; 9.16)	1.89 (1.27; 2.82)	0.63 (0.38; 1.05)

All effects are adjusted for all other sexual behaviours, age and educational level

*AI* anal intercourse, *STI* sexually transmitted infection

^a^(1) and (2) indicate first and second wave within the year respectively

^b^Numbers of casual partners are log transformed in the model

^c^Reference group is ‘no steady partner’

The second multivariable model including data from 2008 onwards (Table 4) showed that recent STI and having chemsex were significantly associated with higher perceived risk from the second half of 2011 until 2016, when risk perception was higher than the grand mean (recent STI aOR 1.36 [95% CI 1.11–1.66]; chemsex aOR 1.29 [95% CI 1.05–1.59]). The addition of PrEP use in the low risk perception period 2017–2018 (third multivariable model) showed that PrEP use was strongly associated with a lower perceived risk of HIV (aOR 0.11 [95% CI 0.06-0.18]).

**Table 4:** Multivariable associations between sexual behaviours, STI at previous visit and risk perception within periods of relatively low and high risk perception between 2008 and 2018

	2008(2)–2011(1)^a^ Low risk perception	2011(2)–2016^a^ High risk perception	2017–2018 Low risk perception
	n visits = 2047	n visits = 4844	n visits = 1768
	OR (95% CI)	OR (95% CI)	OR (95% CI)
Number of casual partners insertive AI^b^	1.28 (1.05; 1.57)	1.30 (1.17; 1.45)	1.28 (1.05; 1.56)
Number of casual partners receptive AI^b^	1.50 (1.22; 1.85)	1.29 (1.15; 1.45)	1.28 (1.06; 1.56)
Condomless AI with casual partner	2.70 (1.91; 3.83)	2.88 (2.38; 3.48)	3.21 (2.19; 4.70)
No condomless AI with steady partner^c^	0.76 (0.52; 1.12)	0.71 (0.56; 0.89)	0.70 (0.45; 1.08)
Condomless AI with steady partner^c^	0.98 (0.67; 1.43)	0.65 (0.53; 0.81)	0.56 (0.38; 0.82)
Condomless receptive AI with a partner living with HIV	1.97 (0.72; 5.38)	1.92 (1.26; 2.92)	0.51 (0.29; 0.90)
AI during group sex	1.44 (0.99; 2.07)	1.01 (0.83; 1.24)	1.20 (0.81; 1.78)
Chemsex	1.31 (0.89; 1.92)	1.29 (1.05; 1.59)	0.95 (0.66; 1.36)
Recent STI	0.81 (0.55; 1.21)	1.36 (1.11; 1.66)	0.82 (0.56; 1.20)

All effects are adjusted for all other sexual behaviours, age and educational level

*AI* anal intercourse, *STI* sexually transmitted infection

^a^(1) and (2) indicate first and second wave within the year respectively

^b^Numbers of casual partners are log transformed in the model

^c^Reference group is ‘no steady partner’

For all multivariable models, the proportional odds assumption was not met. Applying multilevel binary logistic regression models using different thresholds to dichotomize risk perception, yielded similar results (Supplementary tables S2–S3).

## Discussion

Among MSM participating in the Amsterdam Cohort Studies (ACS), we found that perceived risk of HIV has fluctuated at the population level in the past 20 years. Based on relative differences in risk perception between waves and the overall grand mean, we distinguished five alternating periods of relatively low and high perceived risk of HIV. Throughout these time periods, increasing number of receptive and insertive AI partners and having condomless AI with casual partners were consistently associated with higher risk perception. In contrast, condomless receptive AI with a partner living with HIV was no longer associated with perceived HIV risk in 2017–2018. MSM who had condomless AI with a steady partner initially were more likely to have a higher perceived risk, but since 2011 it was associated with lower risk of acquiring HIV.

Our study has major strengths. We used data from the ACS, a prospective cohort study including a large group of MSM with long periods of follow-up. We were able to study the course of risk perception over a time span of 20 years, covering a period in which important developments occurred. Extensive behavioural information was collected in the ACS, allowing us to study the individual contribution of a range of sexual behaviours associated with perceived risk of HIV. This information was reinforced with STI testing results, which were available since 2008. Despite these strengths, our findings must be interpreted in the context of some limitations. In the ACS, highly educated MSM are overrepresented. Additionally, participants were recruited by convenience sampling and chain referral sampling. By participating in a study on HIV, the participants may, by definition, be more aware of risk of acquiring HIV than MSM in the general population. This may limit generalizability to the entire Dutch MSM population. However, behavioural outcomes from the ACS have been previously found to be similar to larger nation-wide monitoring studies, such as the Schorer Monitor [25]. Furthermore, repeatedly filling in questionnaires covering HIV related topics may have raised awareness of developments in the HIV field. The time taken before MSM in the general population become aware of these developments, and their ability to impact the perceived risk of HIV, could be longer. Another possible limitation is that the ACS is an open cohort wherein participants can leave and enter the cohort over time. We cannot completely rule out the influence of the changing study sample on longitudinal changes in risk perception. We attempted to minimize this bias by controlling for age and year of study entry in our analyses. We also reran our analysis among MSM who participated in the cohort for at least 6 years, therefore contributing to both a period of relatively low and relatively high risk perception, and identified the same pattern of change in risk perception. Finally, ordinal logistic regression requires that the effect estimates are equal across categories of risk perception. This proportional odds assumption did not hold for the models presented here, indicating that the strength of the sexual behaviour effect estimates differed across the risk perception scale. However, applying a binary logistic regression approach using different thresholds to dichotomize risk perception [26], resulted in the same conclusions.

The periods of low and high risk perception that we distinguished were based on wave specific differences from the grand mean. Between 1999 and 2008, these periods also included waves in which risk perception did not significantly differ from this mean. From 2008 onwards a clearer pattern arose. Here we describe how these fluctuations from 2008 onwards may be interpreted in light of developments in HIV treatment and prevention and patterns of HIV incidence in the Netherlands, keeping in mind that no conclusions regarding causality can be sufficiently drawn. The low risk perception period between 2008 and 2011 may reflect optimism resulting from the first studies indicating that HIV transmission is low when a partner living with HIV has an undetectable viral load, as was reported in the Swiss statement in 2008 [27]. The high risk perception period between 2011 and 2016 may be related to the high annual numbers of new HIV diagnoses among MSM during these years [22]. This may have raised concerns of HIV risk via widespread media and recognition of more HIV diagnoses within a person’s network. Also, promoting regular testing and the implementation of an opt-out HIV testing policy at all STI clinics in the Netherlands in 2010 [28, 29], together with increasing sexual risk behaviour and increasing STI incidence [18], may have increased risk perception among MSM in this period. In 2017–2018, risk perception was relatively low and might have been related to optimism about the impact of biomedical prevention strategies for HIV, including treatment as prevention and PrEP use. In 2016, PrEP was approved in Europe and the Undetectable equals Untransmittable (U = U) consensus statement was issued [30], indicating that people living with HIV on ART with an undetectable viral load in their blood do not sexually transmit HIV. The U = U statement may have made MSM more confident to rely on undetectable viral load of their partner living with HIV. This is supported by our findings that MSM became more likely to have condomless receptive AI with a partner living with HIV and, in 2017–2018, this did not influence their perceived risk. Since 2015, an increasing number of ACS participants reported PrEP use [24]. We showed that PrEP users in the ACS have a lower perceived risk. Lowered perceived risk of acquiring HIV could also stem from individuals having sex with a person using PrEP [31].

The consistent associations between risk perception and numbers of casual AI partners or having condomless AI with a casual partner, which are well known risk factors for HIV [32], support the hypothesis that MSM have, overall, an adequate risk assessment. The finding that having condomless receptive AI with a partner living with HIV is no longer associated with higher perceived risk could also signify ‘adequate’ risk assessment, under the assumption that MSM only have unprotected sex with partners with an undetectable viral load. We also found that, in 2017–2018, a recent STI was not associated with increased risk perception, which can be considered adequate since with PrEP use and viral load sorting, risk behaviour for a bacterial STI does not necessarily coincide with a high risk for HIV. Strikingly, however, condomless AI with a steady partnership has been related to a lower risk perception since 2011. Studies using data collected before 2011 suggest that the steady partner is an important source of infection, with up to two thirds of infections attributed to sex with a steady partner [33–35]. More recent studies in Western countries suggest a smaller role of the steady partner [36, 37]. An Australian study found that only 10% of recently infected MSM believed they acquired HIV from a steady partner [37]. In the last 15 years, preventive interventions in the Netherlands have promoted negotiated safety, whereby couples agree to always use condoms or PrEP when having AI with casual partners, together with regular HIV testing [38]. Practicing negotiated safety or having a monogamous relationship with an HIV-negative partner may be possible explanations why condomless sex with a steady partner is associated with lower perceived risk. However, MSM who are in a steady partnership and underestimate the risk of their partner’s behaviour may be a target group for prevention.

Currently, we are in a period of low risk perception, most likely influenced by the availability of many risk reduction strategies. If the actual risk of acquiring HIV becomes smaller in coming years, due to successful implementation and scale up of biomedical prevention strategies allowing to reach the 95-95-95 targets set by UNAIDS, risk perception may continue to stay low or decrease even further. Alternatively, low risk perception may make MSM indifferent about acquiring HIV, which may result in lower PrEP uptake, lower adherence, and lower condom use. This could potentially give rise to new HIV infections and subsequently lead to a period of higher risk perception. Monitoring risk perception among MSM will therefore be important for future prevention. To prevent new infections, intervention strategies should target people who underestimate their own risk and continue to engage in risky behaviour. Such approaches should take into account that assessment of HIV risk is complex and condomless AI can be considered as non-risky if biomedical prevention strategies or negotiated safety have been successfully implemented.

Apart from the relationship between individual behaviour and risk perception, which was the focus of the current study, other environmental factors may also influence a person’s risk perception, including attitudes and perceptions of HIV risk within a person’s network and within the larger societal context. Future research may focus on these environmental factors. Another direction for future research may be to examine the mediating role of risk perception in the association between sexual behaviour and risk reduction strategies. In studying this mediating role, factors that may limit risk reduction behaviour despite adequate risk perception will need consideration, including stigma, social barriers, social inequality and social disparity.

In conclusion, our study showed that HIV risk perception among MSM has fluctuated in the past 20 years. U = U and PrEP use may have resulted in lower perceived risk in 2017–2018. As long as MSM adhere to current HIV risk reduction strategies, including condom use, regular HIV testing, viral load sorting, and use of PrEP, low risk perception is in agreement with their actual risk. Awareness of MSM who underestimate their risk and become careless in taking preventive measures is needed to stop further transmission.

## Electronic supplementary material

Below is the link to the electronic supplementary material.

Supplementary file1 (PDF 159 KB)

## References

[1] Biello KB, Edeza A, Montgomery MC, Almonte A, Chan PA (2019). Risk perception and interest in HIV pre-exposure prophylaxis among men who have sex with men with rectal gonorrhea and chlamydia infection. Arch Sex Behav.

[2] Peng P, Su S, Fairley CK (2018). A global estimate of the acceptability of pre-exposure prophylaxis for HIV among men who have sex with men: a systematic review and meta-analysis. AIDS Behav.

[3] Chan PA, Glynn TR, Oldenburg CE (2016). Implementation of preexposure prophylaxis for human immunodeficiency virus prevention among men who have sex with men at a New England sexually transmitted diseases clinic. Sex Transm Dis.

[4] Arrington-Sanders R, Wilson CM, Perumean-Chaney SE, Patki A, Hosek S (2018). The role of socio-behavioral factors in sub-protective tenofovir diphosphate (TFV-DP) levels among YMSM enrolled in two PrEP trials. J Acquir Immune Defic Syndr.

[5] Clifton S, Nardone A, Field N (2016). HIV testing, risk perception, and behaviour in the British population. AIDS.

[6] Evangeli M, Pady K, Wroe AL (2016). Which psychological factors are related to HIV testing? A quantitative systematic review of global studies. AIDS Behav.

[7] Wilton J, Kain T, Fowler S (2016). Use of an HIV-risk screening tool to identify optimal candidates for PrEP scale-up among men who have sex with men in Toronto, Canada: disconnect between objective and subjective HIV risk. J Int AIDS Soc.

[8] Deblonde J, De Koker P, Hamers FF, Fontaine J, Luchters S, Temmerman M (2010). Barriers to HIV testing in Europe: a systematic review. Eur J Public Health.

[9] Marcus U, Gassowski M, Drewes J (2016). HIV risk perception and testing behaviours among men having sex with men (MSM) reporting potential transmission risks in the previous 12 months from a large online sample of MSM living in Germany. BMC Public Health.

[10] Hachfeld A, Ledergerber B, Darling K (2015). Reasons for late presentation to HIV care in Switzerland. J Int AIDS Soc.

[11] Kesler MA, Kaul R, Liu J (2016). Actual sexual risk and perceived risk of HIV acquisition among HIV-negative men who have sex with men in Toronto, Canada. BMC Public Health.

[12] Evangeli M, Baker LL, Pady K, Jones B, Wroe AL (2016). What leads some people to think they are HIV-positive before knowing their diagnosis? A systematic review of psychological and behavioural correlates of HIV-risk perception. AIDS Care.

[13] Jansen IA, Geskus RB, Davidovich U (2011). Ongoing HIV-1 transmission among men who have sex with men in Amsterdam: a 25-year prospective cohort study. AIDS.

[14] Katz MH, Schwarcz SK, Kellogg TA (2002). Impact of highly active antiretroviral treatment on HIV seroincidence among men who have sex with men: San Francisco. Am J Public Health.

[15] Rietmeijer CA, Patnaik JL, Judson FN, Douglas JM (2003). Increases in gonorrhea and sexual risk behaviors among men who have sex with men: a 12-year trend analysis at the Denver Metro Health Clinic. Sex Transm Dis.

[16] Kalichman SC, Price D, Eaton LA (2017). Diminishing perceived threat of AIDS and increasing sexual risks of HIV among men who have sex with men, 1997–2015. Arch Sex Behav.

[17] Chen YH, Snowden JM, McFarland W, Raymond HF (2016). Pre-exposure prophylaxis (PrEP) use, seroadaptation, and sexual behavior among men who have sex with men, San Francisco, 2004–2014. AIDS Behav.

[18] van Bilsen WPH, Boyd A, van der Loeff MFS (2020). Diverging trends in incidence of HIV versus other sexually transmitted infections in HIV-negative men who have sex with men (MSM) in Amsterdam. AIDS.

[19] Unemo M, Bradshaw CS, Hocking JS (2017). Sexually transmitted infections: challenges ahead. Lancet Infect Dis.

[20] Bezemer D, de Wolf F, Boerlijst MC (2008). A resurgent HIV-1 epidemic among men who have sex with men in the era of potent antiretroviral therapy. AIDS.

[21] Sullivan PS, Hamouda O, Delpech V (2009). Reemergence of the HIV epidemic among men who have sex with men in North America, Western Europe, and Australia, 1996–2005. Ann Epidemiol.

[22] van Sighem AI, Boender TS, Wit FWNM, Smit C, Matser A, Reiss P. Monitoring Report 2018. Human immunodeficiency virus (HIV) infection in the Netherlands. Amsterdam: Stichting HIV Monitoring; 2018

[23] van Griensven GJ, de Vroome EM, Goudsmit J, Coutinho RA (1989). Changes in sexual behaviour and the fall in incidence of HIV infection among homosexual men. BMJ.

[24] Coyer L, van Bilsen W, Bil J (2018). Pre-exposure prophylaxis among men who have sex with men in the Amsterdam Cohort Studies: use, eligibility, and intention to use. PLoS One.

[25] Van Empelen P, Van Berkel M, Roos E, Zuilhof W (2011). Schorer Monitor.

[26] Bender R, Grouven U (1998). Using binary logistic regression models for ordinal data with non-proportional odds. J Clin Epidemiol.

[27] Vernazza P, Hirschel B, Bernasconi E, Flepp M (2008). Les personnes séropositives ne souffrant d’aucune autre MST et suivant un traitment antirétroviral efficace ne transmettent pas le VIH par voie sexuelle. Bull Med Suisses.

[28] Heijman RL, Stolte IG, Thiesbrummel HF (2009). Opting out increases HIV testing in a large sexually transmitted infections outpatient clinic. Sex Transm Infect.

[29] Dukers-Muijrers NH, Niekamp AM, Vergoossen MM, Hoebe CJ (2009). Effectiveness of an opting-out strategy for HIV testing: evaluation of 4 years of standard HIV testing in a STI clinic. Sex Transm Infect.

[30] Brady M, Cohen M, Daskalakis DC, et al. Consensus Statement [Internet]. Prevention Access Campaign; [updated 2019 May 5; cited 2020 March 26]. https://www.preventionaccess.org/consensus.

[31] Martinez JE, Jonas KJ (2019). Pre-exposure prophylaxis sorting among men who have sex with men. AIDS Care.

[32] Basten M, Heijne JCM, Geskus R, Den Daas C, Kretzschmar M, Matser A (2018). Sexual risk behaviour trajectories among MSM at risk for HIV in Amsterdam, the Netherlands. AIDS.

[33] Davidovich U, de Wit J, Albrecht N, Geskus R, Stroebe W, Coutinho R (2001). Increase in the share of steady partners as a source of HIV infection: a 17-year study of seroconversion among gay men. AIDS.

[34] Sullivan PS, Salazar L, Buchbinder S, Sanchez TH (2009). Estimating the proportion of HIV transmissions from main sex partners among men who have sex with men in five US cities. AIDS.

[35] Xiridou M, Geskus R, De Wit J, Coutinho R, Kretzschmar M (2003). The contribution of steady and casual partnerships to the incidence of HIV infection among homosexual men in Amsterdam. AIDS.

[36] Hansson D, Leung KY, Britton T, Stromdahl S (2019). A dynamic network model to disentangle the roles of steady and casual partners for HIV transmission among MSM. Epidemics.

[37] Down I, Ellard J, Bavinton BR, Brown G, Prestage G (2017). In Australia, most HIV infections among gay and bisexual men are attributable to sex with ‘new’ partners. AIDS Behav.

[38] Davidovich U, De Wit JBF, Stroebe W. Using the Internet to reduce risk of HIV-Infection in steady relationships: a randomized controlled trial of a tailored intervention for gay men. In Thesis: Liaisons dangereuses: HIV risk behavior and prevention in steady gay relationships. Davidovich U [author]. Amsterdam; 2006. pp. 96–120.

