# Supplementary material for: The Rhythm of Risk: Sexual Behaviour, PrEP Use and HIV Risk Perception Between 1999 and 2018 Among Men Who Have Sex with Men in Amsterdam, The Netherlands
Source: AIDS Behav. 2020 Dec 2;25(6):1800–9. doi: 10.1007/s10461-020-03109-4 (PMC8081694; doi:10.1007/s10461-020-03109-4)
Supplement: Supplementary file 1 — Supplementary file1 (PDF 159 KB) [file 10461_2020_3109_MOESM1_ESM.pdf]

Supplementary materials

Title: The rhythm of risk: Sexual behaviour, PrEP use and HIV risk perception between 1999 and 2018 among men who have sex with men in Amsterdam, the Netherlands

Authors: Maartje Basten, Chantal den Daas, Janneke C.M. Heijne, Anders Boyd, Udi Davidovich, Ganna Rozhnova, Mirjam Kretzschmar and Amy Matser

Journal: AIDS and Behavior

Supplementary table S1 Changes in behaviour assessment in the Amsterdam Cohort Studies between 1999 and 2018

Variable	Assessment across time
Risk perception	1999-2018: Participants were asked to rate the likelihood that they acquired HIV in the preceding six months, defined herein as their "perceived HIV risk". This question was answered on a 7-point Likert scale from 1 being 'impossible' to 7 being 'very likely'
Number of casual partners insertive and receptive AI	1999 – 2007 (1)*: Participants were asked to report the total number of casual partners with whom they had insertive and receptive AI in the past six months.<br>2007 (2) – 2017 (1)*: Participants were asked to report the number of casual partners with whom they had insertive and receptive AI in the past six months for three subtypes: one-night stands, multiple-time casual partners and sex buddies. These three types were added up to get the total number of casual partners.<br>2017 (2) – 2018*: Participants were asked to report the number of casual partners with whom they had insertive and receptive AI in the past six months for two subtypes: anonymous partners and known partners. These two types were added up to get the total number of casual partners
Condomless AI with a casual partner (yes/no)	1999 – 2007 (1)*: Participants were asked how often they used a condom when having insertive or receptive AI with casual partners in the past six months. These questions were answered on a 5-point Likert scale from 1 'always' to 5 'never'. If a person did not always use a condom it was scored as having had condomless AI with a casual partner.<br>2007 (2) – 2017 (1)*: Participants were asked how often they used a condom when having insertive or receptive AI for three types of casual partners: one-night stands, multiple-time casual partners and sex buddies. If a person did not always use a condom with one of these casual partners it was scored as having had condomless AI with a casual partner.<br>2017 (2) – 2018*: Participants were asked whether they had condomless insertive and receptive AI with anonymous and known casual partners in the past six months (yes/no).
Condomless AI with a steady partner, 3 categories:<br>1) no steady partner;<br>2) no condomless AI with steady partner;<br>3) condomless AI with steady partner	1999 – 2018: Participants were asked if they had a steady relationship with a man in the past six months. Subsequently they were asked if they had insertive and receptive AI with their steady partner and how often they had used a condom (5-point Likert scale from 1 'always' to 5 'never'). If a person had a steady partner but did not have AI or always used a condom during AI it was scored as 'no condomless AI with steady partner'. If a person had AI with his steady partner and did not always use a condom it was scored as 'condomless AI with steady partner'.

Supplementary table S1: continued

Variable	Assessment across time
Condomless receptive AI with a partner living with HIV (yes/no)	For steady partners:<br>2002 - 2018: Participants were asked if they knew the HIV-status of their steady partner. This information was combined with information on receptive AI and condom use with their steady partner (see above) to define whether the participant had condomless receptive AI with a steady partner living with HIV.<br>For casual partners:<br>2002 - 2007(1)*: Participants were asked whether they had unprotected receptive AI with a casual partner living with HIV.<br>2007(2) – 2017(1)*: Participants were asked whether they had unprotected receptive AI with a casual partner living with HIV for three types of casual partners: one-night stands, multiple-time casual partners and sex buddies.<br>2017(2) – (2018)*: Participants who reported to have had condomless receptive AI with anonymous or known casual partners were asked how many of these partners were living with HIV and whether they had a detectable, undetectable or unknown viral load. Information on viral load status was ignored for this variable.<br>Information on steady partner and casual partners was combined to define whether participants had condomless receptive AI with a partner living with HIV.
AI during group sex (yes/no)	2008 - 2015 (1)*: Participants were asked if they had insertive or receptive AI with a casual partner during group sex in the past six months. Group sex included sex with at least three persons.<br>2015 (2) -2018*: Participants were asked if they participated in group sex in the past six months. Group sex included sex with at least three persons. Subsequently, they were asked if they had insertive or receptive AI during the last time they had group sex.
Chemsex (yes/no)	2008 – 2018: Participants were asked if they used different types of drugs in the past six months and in which context they used each type of drugs. Context was divided into: 'in general', 'sex with steady partner', 'sex with casual partners' and 'group sex'. Chemsex was defined as using GBL, GHB, mephedrone, methamphetamine, ketamine, amphetamine, cocaine, or XTC during sex with steady partner, casual partners or during group sex.

Note. AI= anal intercourse; * (1) and (2) indicate first and second wave within the year respectively.

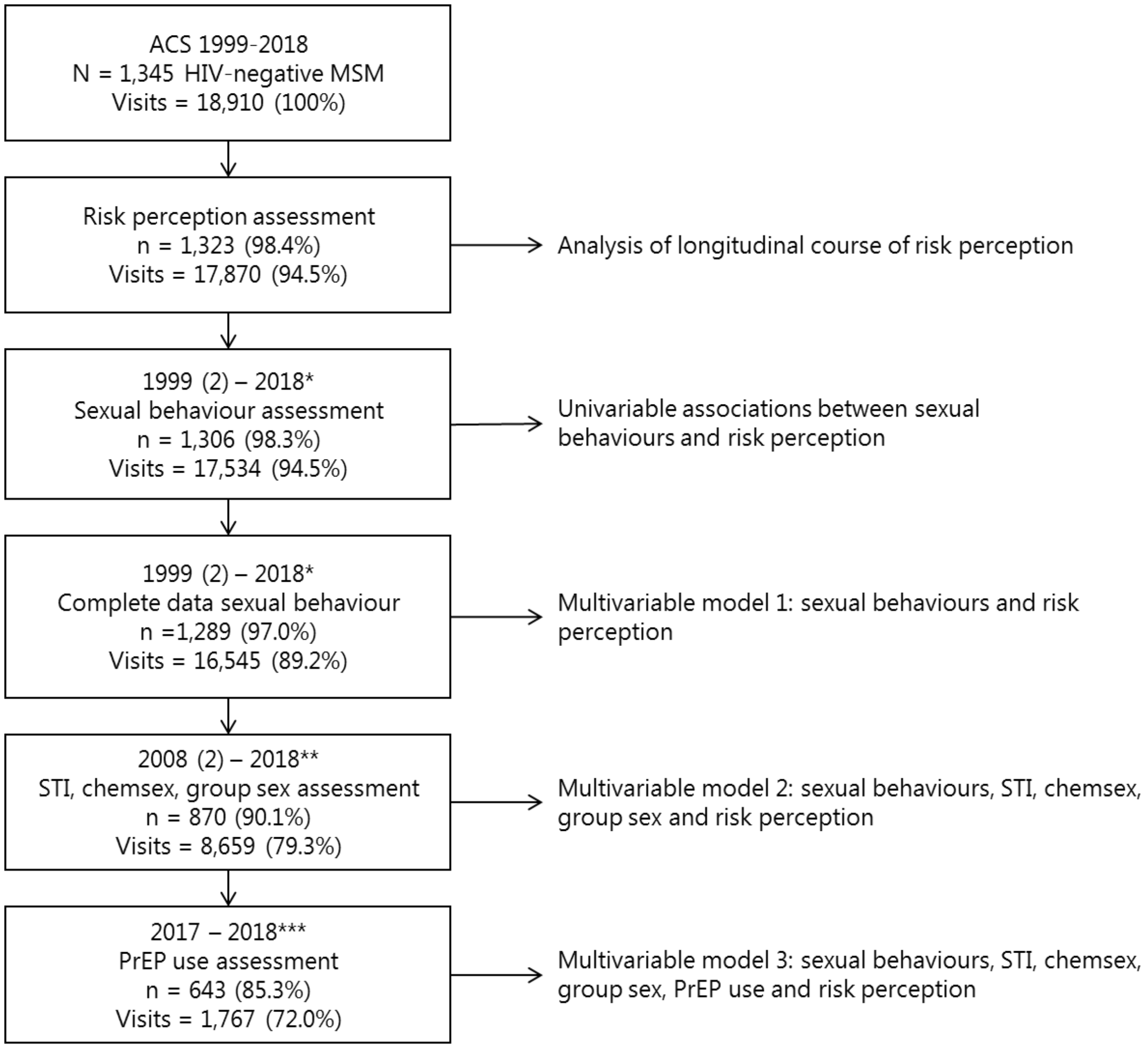

Supplementary figure S1 Study population flow chart

Note. * Percentages based on all HIV-negative MSM visits from second half of 1999 onwards: n = 1,329, visits = 18,556; ** Percentages based on all HIV-negative MSM visits from second half of 2008 onwards: n = 966, visits = 10,925; *** Percentages based on all HIV-negative MSM visits from 2017 onwards: n = 754, visits = 2,455

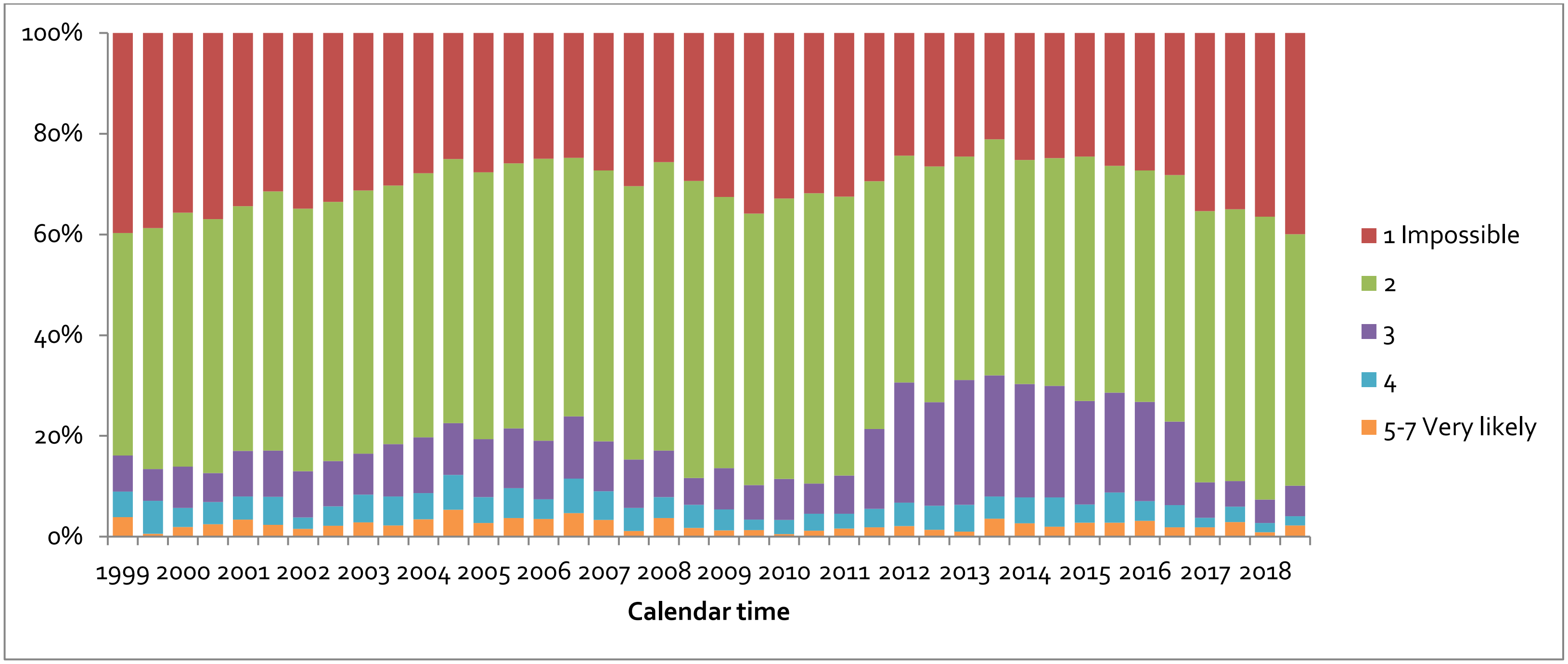

Supplementary figure S2 Distribution of risk perception score at each biannual wave from 1999 to 2018 among 1,323 MSM (n=17,870 visits).

Note. MSM were asked to rate the likelihood that they acquired HIV in the preceding 6 months. This question was answered on a 7-point Likert scale from 1 being 'impossible' to 7 being 'very likely'. Because few MSM responded with high perceived risk , we combined answer categories 5 through 7 into one category, resulting in 5 levels of risk perception.

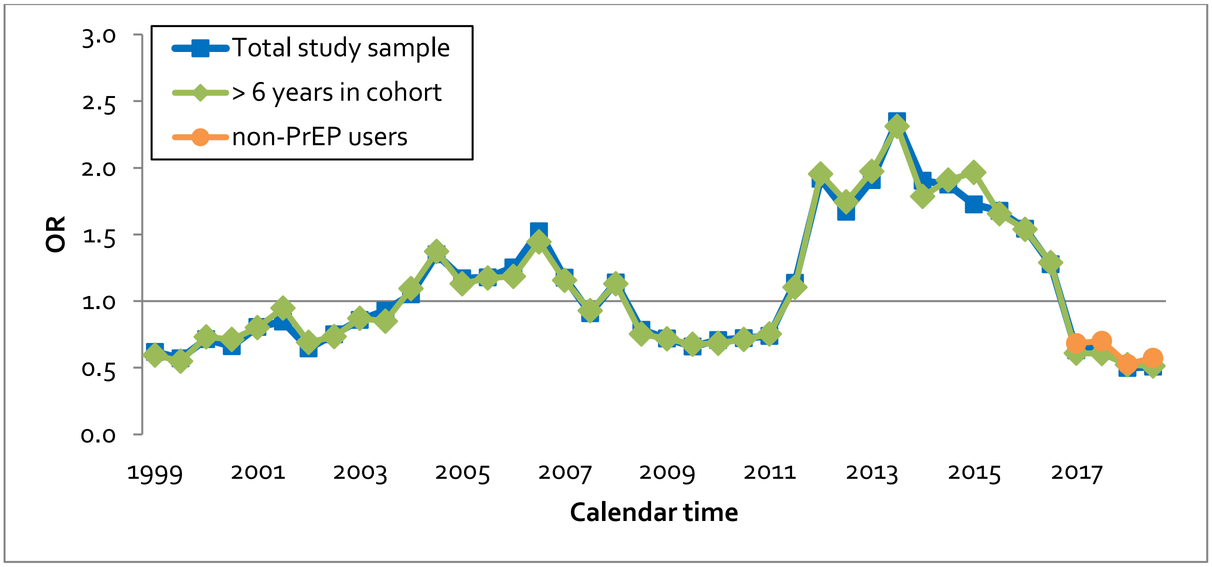

Supplementary figure S3: Relative differences in risk perception score across time in the total sample (blue; n=1,309 MSM, 17,811 visits), in MSM who participated > six years in the study (green; n=570 MSM, 14,083 visits) and in non-PrEP users in 2017-2018 (orange; n=677 MSM, 1,971 visits)

Note. Relative differences are presented as OR's for each wave compared to the grand mean (i.e. the distribution of the risk perception score across all visits). OR's below one indicate lower odds for a one-point higher score on the risk perception scale, while OR's above one indicate higher odds for a one-point higher score on the risk perception scale. OR's are adjusted for age, year of study entry and education level.

Supplementary Table S2 Multivariable associations between sexual behaviours and risk perception within periods of relatively low and high risk perception between 1999-2018 based on mixed effects binary logistic regression, using different cut-offs of risk perception score.

	1999 (2) - 2003 [a] **LOW RISK PERCEPTION** n visits = 3,137	2004 - 2008 (1) [a] **HIGH RISK PERCEPTION** n visits = 3,614	2008 (2) - 2011 (1) [a] **LOW RISK PERCEPTION** n visits = 2,269	2011 (2) - 2016 [a] **HIGH RISK PERCEPTION** n visits = 5,373	2017 - 2018 **LOW RISK PERCEPTION** n visits = 2,152
	OR (95%CI)	OR (95%CI)	OR (95%CI)	OR (95%CI)	OR (95%CI)
**Risk perception score 1 (ref) vs. 2-5**					
Number of casual partners insertive AI [b]	1.52 (1.17;1.98)	2.07 (1.65; 2.60)	1.48 (1.15; 1.91)	1.39 (1.19; 1.63)	1.36 (1.10; 1.69)
Number of casual partners receptive AI [b]	2.48 (1.89; 3.25)	1.88 (1.48; 2.38)	2.09 (1.58; 2.76)	1.68 (1.41; 2.01)	1.44 (1.15; 1.81)
Condomless AI with casual partner	3.99 (2.67; 6.20)	3.48 (2.28; 5.32)	2.12 (1.33; 3.38)	3.79 (2.76; 5.22)	3.17 (2.02; 4.96)
No condomless AI with steady partner [c]	1.45 (1.01; 2.07)	1.23 (0.86; 1.75)	0.82 (0.52; 1.30)	0.75 (0.55; 1.02)	0.80 (0.49; 1.31)
Condomless AI with steady partner [c]	1.61 (1.13; 2.31)	1.25 (0.89; 1.75)	1.03 (0.65; 1.62)	0.73 (0.55; 0.97)	0.59 (0.38; 0.91)
Condomless receptive AI with partner living with HIV	-	2.11 (0.51; 8.73)	3.80 (0.62; 23.33)	1.37 (0.66; 2.84)	0.33 (0.17; 0.63)
**Risk perception score 1-2 (ref) vs. 3-5**					
Number of casual partners insertive AI b	1.54 (1.23;1.93)	1.37 (1.14; 1.64)	1.20 (0.92; 1.56)	1.22 (1.08; 1.37)	1.08 (0.86; 1.35)
Number of casual partners receptive AI b	1.41 (1.14; 1.75)	1.67 (1.38; 2.01)	1.35 (1.03; 1.76)	1.24 (1.09; 1.41)	1.09 (0.87; 1.38)
Condomless AI with casual partner	5.97 (4.20; 8.50)	5.21 (3.84; 7.08)	4.79 (2.97; 7.73)	2.68 (2.16; 3.33)	3.58 (2.19; 5.85)
No condomless AI with steady partner c	1.20 (0.82; 1.76)	0.87 (0.61; 1.24)	0.68 (0.38; 1.20)	0.58 (0.44; 0.76)	0.87 (0.49; 1.52)
Condomless AI with steady partner c	1.50 (1.05; 2.14)	1.11 (0.80; 1.54)	1.11 (0.66; 1.88)	0.63 (0.50; 0.81)	0.58 (0.36; 0.96)
Condomless receptive AI with partner living with HIV	-	8.59 (3.16; 23.36)	4.37 (1.46; 13.04)	1.82 (1.14; 2.91)	0.94 (0.46; 1.92)

Supplementary table S2 continued

	1999 (2) - 2003 a **LOW RISK PERCEPTION**	2004 - 2008 (1) a **HIGH RISK PERCEPTION**	2008 (2) - 2011 (1) a **LOW RISK PERCEPTION**	2011 (2) - 2016 a **HIGH RISK PERCEPTION**	2017 - 2018 **LOW RISK PERCEPTION**
	OR (95%CI)	OR (95%CI)	OR (95%CI)	OR (95%CI)	OR (95%CI)
**Risk perception score 1-3 (ref) vs. 4-5**					
Number of casual partners insertive AI [b]	1.46 (1.11;1.93)	1.56 (1.24; 1.96)	1.22 (0.87; 1.70)	1.13 (0.95; 1.35)	1.18 (0.88; 1.59)
Number of casual partners receptive AI [b]	1.54 (1.18; 2.00)	1.54 (1.23; 1.93)	1.06 (0.75; 1.48)	1.24 (1.03; 1.49)	1.08 (0.80; 1.45)
Condomless AI with casual partner	5.57 (3.53; 8.77)	5.19 (3.56; 7.56)	5.69 (3.05; 10.63)	6.19 (4.33; 8.85)	2.86 (1.46; 5.60)
No condomless AI with steady partner [c]	1.53 (0.91; 2.56)	1.20 (0.75; 1.93)	0.76 (0.34; 1.68)	0.61 (0.39; 0.96)	0.52 (0.23; 1.16)
Condomless AI with steady partner [c]	1.83 (1.14; 2.93)	1.39 (0.90; 2.12)	1.29 (0.66; 2.54)	0.49 (0.33; 0.72)	0.69 (0.37; 1.31)
Condomless receptive AI with partner living with HIV	-	9.74 (3.59; 26.46)	5.00 (1.50; 16.70)	3.19 (1.77; 5.74)	1.54 (0.65; 3.64)
**Risk perception score 1-4 (ref) vs. 5**					
Number of casual partners insertive AI b	1.49 (1.02; 2.17)	1.57 (1.20; 2.05)	1.64 (0.83; 3.25)	1.21 (0.94; 1.58)	1.06 (0.71; 1.57)
Number of casual partners receptive AI b	1.44 (1.02; 2.03)	1.46 (1.13; 1.90)	1.24 (0.67; 2.31)	1.00 (0.76; 1.32)	1.03 (0.68; 1.54)
Condomless AI with casual partner	5.73 (3.06; 10.74)	3.20 (1.95; 5.28)	3.67 (1.12; 11.97)	4.65 (2.68; 8.06)	2.00 (0.79; 5.07)
No condomless AI with steady partner c	1.76 (0.84; 3.69)	1.18 (0.64; 2.18)	1.28 (0.22; 7.47)	0.71 (0.37; 1.36)	0.54 (0.18; 1.57)
Condomless AI with steady partner c	1.60 (0.82; 3.14)	1.21 (0.70; 2.11)	4.36 (1.08; 17.60)	0.60 (0.34; 1.08)	0.68 (0.29; 1.59)
Condomless receptive AI with partner living with HIV	-	14.37 (5.20; 39.73)	24.64 (3.76; 161.30)	3.64 (1.68; 7.88)	2.89 (0.98; 8.53)

Note. All effects are adjusted for all other sexual behaviours, age and educational level. AI=anal intercourse; STI= sexually transmitted infection. [a] (1) and (2) indicate first and second wave within the year respectively. [b] Numbers of casual partners are log transformed in the model. [c] Reference group is 'no steady partner'.

Supplementary Table S3 Multivariable associations between sexual behaviours, STI at previous visit and risk perception within periods of relatively low and high risk perception between 2008-2018 based on mixed effects binary logistic regression, using different cut-offs of risk perception score.

	2008 (2) - 2011 (1) [a] **LOW RISK PERCEPTION** n visits = 2,047	2011 (2) - 2016 [a] **HIGH RISK PERCEPTION** n visits = 4,844	2017 - 2018 [a] **LOW RISK PERCEPTION** n visits = 1,768
	OR (95%CI)	OR (95%CI)	OR (95%CI)
**Risk perception score 1 (ref) vs. 2-5**			
Number of casual partners insertive AI [b]	1.35 (1.02; 1.78)	1.42 (1.19; 1.70)	1.40 (1.08; 1.81)
Number of casual partners receptive AI [b]	1.82 (1.35; 2.46)	1.65 (1.35; 2.00)	1.43 (1.10; 1.86)
Condomless AI with casual partner	2.25 (1.37; 3.69)	3.68 (2.61; 5.19)	3.60 (2.14; 6.05)
No condomless AI with steady partner [c]	0.72 (0.44; 1.17)	0.78 (0.55; 1.09)	0.68 (0.39; 1.19)
Condomless AI with steady partner [c]	0.87 (0.53; 1.43)	0.65 (0.47; 0.89)	0.56 (0.34; 0.93)
Condomless receptive AI with a partner living with HIV	2.01 (0.31; 12.92)	1.22 (0.56; 2.65)	0.24 (0.12; 0.51)
AI during group sex	1.90 (1.12; 3.24)	1.01 (0.72; 1.43)	1.36 (0.80; 2.32)
Chemsex	1.23 (0.74; 2.05)	1.31 (0.96; 1.81)	0.79 (0.50; 1.27)
Recent STI	0.80 (0.46; 1.41)	2.08 (1.42; 3.04)	0.74 (0.45; 1.22)
**Risk perception score 1-2 (ref) vs. 3-5**			
Number of casual partners insertive AI b	1.23 (0.91; 1.65)	1.22 (1.07; 1.40)	1.00 (0.76; 1.31)
Number of casual partners receptive AI b	1.39 (1.03; 1.86)	1.18 (1.03; 1.36)	1.16 (0.89; 1.52)
Condomless AI with casual partner	4.79 (2.88; 7.96)	2.60 (2.06; 3.29)	4.40 (2.48; 7.81)
No condomless AI with steady partner c	0.66 (0.36; 1.21)	0.61 (0.46; 0.81)	0.78 (0.41; 1.48)
Condomless AI with steady partner c	1.09 (0.62; 1.91)	0.58 (0.45; 0.76)	0.50 (0.28; 0.89)
Condomless receptive AI with a partner living with HIV	2.13 (0.61; 7.52)	2.02 (1.23; 3.32)	0.96 (0.44; 2.10)
AI during group sex	0.97 (0.54; 1.71)	1.03 (0.80; 1.34)	1.01 (0.56; 1.83)
Chemsex	1.11 (0.63; 1.95)	1.21 (0.93; 1.56)	1.28 (0.75; 2.20)
Recent STI	0.80 (0.43; 1.51)	1.16 (0.90; 1.51)	0.93 (0.52; 1.67)

Supplementary table S3 continued

	2008 (2) - 2011 (1) [a] LOW RISK PERCEPTION	2011 (2) - 2016 [a] HIGH RISK PERCEPTION	2017 - 2018 [a] LOW RISK PERCEPTION
	OR (95%CI)	OR (95%CI)	OR (95%CI)
**Risk perception score 1-3 (ref) vs. 4-5**			
Number of casual partners insertive AI [b]	1.12 (0.76; 1.66)	1.10 (0.90; 1.35)	1.08 (0.77; 1.53)
Number of casual partners receptive AI [b]	1.12 (0.76; 1.64)	1.21 (0.98; 1.48)	1.15 (0.82; 1.60)
Condomless AI with casual partner	4.83 (2.47; 9.45)	5.58 (3.80; 8.20)	3.69 (1.74; 7.86)
No condomless AI with steady partner [c]	0.83 (0.36; 1.93)	0.63 (0.39; 1.01)	0.43 (0.17; 1.09)
Condomless AI with steady partner [c]	1.26 (0.60; 2.64)	0.47 (0.31; 0.73)	0.63 (0.31; 1.27)
Condomless receptive AI with a partner living with HIV	2.39 (0.49; 11.54)	3.48 (1.87; 6.49)	1.17 (0.46; 2.99)
AI during group sex	0.75 (0.34; 1.63)	1.00 (0.67; 1.50)	1.19 (0.56; 2.52)
Chemsex	1.87 (0.91; 3.82)	1.35 (0.91; 1.99)	1.53 (0.77; 3.06)
Recent STI	0.65 (0.26; 1.59)	1.25 (0.86; 1.84)	0.61 (0.27; 1.38)
**Risk perception score 1-4 (ref) vs. 5**			
Number of casual partners insertive AI b	1.16 (0.46; 2.93)	1.19 (0.88; 1.59)	1.09 (0.69; 1.71)
Number of casual partners receptive AI b	1.63 (0.67; 3.93)	0.97 (0.72; 1.31)	1.06 (0.68; 1.64)
Condomless AI with casual partner	2.44 (0.54; 10.93)	4.03 (2.24; 7.26)	2.34 (0.82; 6.63)
No condomless AI with steady partner c	1.41 (0.14; 14.29)	0.62 (0.31; 1.27)	0.39 (0.11; 1.34)
Condomless AI with steady partner c	8.19 (0.93; 71.72)	0.56 (0.30; 1.06)	0.42 (0.16; 1.13)
Condomless receptive AI with a partner living with HIV	10.75 (0.63; 183.78)	3.62 (1.59; 8.26)	1.86 (0.57; 6.04)
AI during group sex	2.13 (0.32; 14.24)	0.95 (0.51; 1.76)	1.42 (0.52; 3.88)
Chemsex	1.09 (0.21; 5.73)	1.34 (0.75; 2.40)	1.66 (0.66; 4.22)
Recent STI	0.16 (0.01; 2.94)	1.44 (0.81; 2.56)	0.74 (0.25; 2.16)

Note. All effects are adjusted for all other sexual behaviours, age and educational level. AI=anal intercourse; STI = sexually transmitted infection. [a] (1) and (2) indicate first and second wave within the year respectively. [b] Numbers of casual partners are log transformed in the model. [c] Reference group is 'no steady partner'.
